# Effect of very low-intensity resistance exercise with slow movement and tonic force generation on post-exercise inhibitory control

**DOI:** 10.1016/j.heliyon.2021.e06261

**Published:** 2021-02-18

**Authors:** Kento Dora, Tadashi Suga, Keigo Tomoo, Takeshi Sugimoto, Ernest Mok, Hayato Tsukamoto, Shingo Takada, Takeshi Hashimoto, Tadao Isaka

**Affiliations:** a Faculty of Sport and Health Science, Ritsumeikan University, Kusatsu, Shiga, Japan; b Faculty of Lifelong Sport, Department of Sports Education, Hokusho University, Ebetsu, Hokkaido, Japan

**Keywords:** Cognitive function, Brain health, Lactate, Electromyographic activity, Arousal

## Abstract

**Background:**

The extremely low loads (e.g., <30% of one-repetition maximum) involved in performing resistance exercise are effective in preventing musculoskeletal injury and enhancing exercise adherence in various populations, especially older individuals and patients with chronic diseases. Nevertheless, long-term intervention using this type of protocol is known to have little effects on muscle size and strength adaptations. Despite this knowledge, very low-intensity resistance exercise (VLRE) with slow movement and tonic force generation (ST) significantly increases muscle size and strength. To further explore efficacy of ST-VLRE in the clinical setting, this study examined the effect of ST-VLRE on post-exercise inhibitory control (IC).

**Methods:**

Twenty healthy, young males (age: 21 ± 0 years, body height: 173.4 ± 1.2 cm, body weight: 67.4 ± 2.2 kg) performed both ST-VLRE and normal VLRE in a crossover design. The load for both protocols was set at 30% of one-repetition maximum. Both protocols were programmed with bilateral knee extension for six sets with ten repetitions per set. The ST-VLRE and VLRE were performed with slow (3-sec concentric, 3-sec eccentric, and 1-sec isometric actions with no rest between each repetition) and normal contractile speeds (1-sec concentric and 1-sec eccentric actions and 1-sec rests between each repetition), respectively. IC was assessed using the color-word Stroop task at six time points: baseline, pre-exercise, immediate post-exercise, and every 10 min during the 30-min post-exercise recovery period.

**Results:**

The reverse-Stroop interference score, a parameter of IC, significantly decreased immediately after both ST-VLRE and VLRE compared to that before each exercise (decreasing rate >32 and 25%, respectively, vs. baseline and/or pre-exercise for both protocols; all *P*s < 0.05). The improved IC following ST-VLRE, but not following VLRE, remained significant until the 20-min post-exercise recovery period (decreasing rate >48% vs. baseline and pre-exercise; both *P*s < 0.001). The degree of post-exercise IC improvements was significantly higher for ST-VLRE than for VLRE (*P* = 0.010 for condition × time interaction effect).

**Conclusions:**

These findings suggest that ST-VLRE can improve post-exercise IC effectively. Therefore, ST-VLRE may be an effective resistance exercise protocol for improving cognitive function.

## 1 Introduction

Resistance exercise is the most beneficial strategy for increasing skeletal muscle size and strength [1, 2]. Recently, long-term intervention of resistance exercise has been shown to improve cognitive function in various populations, including older individuals and patients with chronic diseases [3, 4]. Therefore, resistance exercise is recognized as an effective strategy for enhancing skeletal muscle and cognitive health.

Cognitive inhibitory control (IC) is defined as the suppression of behavior in response to either internal or external stimuli [5], which is necessary to prevent the implementation of an unrequired action [6]. An acute bout of certain resistance exercise protocols, in particular those with high-loads, improves post-exercise IC [7, 8, 9, 10, 11]. The degree to which cognitive function is improved by acute exercise may be associated with the improvement induced by long-term intervention, potentially through amelioration of the brain system (i.e., brain network connectivity) [12]. Therefore, clarifying the degree of acute resistance exercise-induced improvements in cognitive function may be useful for creating an effective resistance exercise program to improve cognitive function, including IC.

In addition to high-intensity resistance exercise (HRE), post-exercise IC can also be improved by low-intensity resistance exercise (LRE) [7, 8, 11]. LRE has a broader applicability than HRE, due to the lower loads exerted on certain physical systems (e.g., cardiovascular and musculoskeletal systems) in particular populations, especially in older individuals and patients with chronic diseases [2, 13, 14, 15]. Furthermore, the low-load involved in performing resistance exercise may assist in increasing exercise adherence in these populations [16]. However, it is well known that long-term HRE increases skeletal muscle size and strength more than long-term LRE [1, 17, 18]. Additionally, we and others previously determined that immediate post-exercise improvement of IC was greater following HRE than LRE [7, 8, 11]. This suggests that conventional LRE may be inadequate in improving IC. Therefore, it would be useful to identify effective strategies to enhance LRE-induced IC improvement.

The exercise intensity involved in performing resistance exercise is a major variable for determining muscle size and strength adaptations induced by long-term resistance exercise [1, 17, 18]. Additionally, movement velocity when resistance exercise is performed is an important variable for these muscle adaptations [19, 20, 21, 22, 23, 24]. Movement velocities during normal resistance exercise are generally prescribed by performing concentric and eccentric actions for 1–2 s [1]. Compared to this normal protocol, specific movement velocity protocols are performed with faster or slower velocities during concentric and eccentric actions [19, 20, 21, 22, 23, 24]. Of those, a typical protocol of slow movement and tonic force generation (ST) is prescribed by performing 3-sec concentric, 3-sec eccentric, and 1-sec isometric actions with no rest between each repetition [20, 21, 22, 23, 24].

Tanimoto et al. [21] reported that long-term intervention of LRE, comprising of 50% of one-repetition maximum (1-RM) with ST, significantly increased muscle size and strength in healthy young individuals. Furthermore, these skeletal muscle adaptations were similar to those induced by long-term HRE (i.e., 80% 1-RM) with normal movement (i.e., 1-sec concentric and 1-sec eccentric actions and 1-sec rests between each repetition) [21, 22]. In a subsequent study, Watanabe et al. [23] reported that long-term intervention of very LRE (VLRE), comprising of 30% 1-RM with ST, significantly increased muscle size and strength in healthy older individuals, whereas no such effects were observed with long-term VLRE with normal movement. Decreasing the exercise intensity of resistance exercise may be useful in mitigating the loads on some physical systems and enhancing exercise adherence [2, 13, 14, 15, 16]. Additionally, to obtain significant muscle size and strength adaptations, training frequency is generally recommended for a minimum of 2–3 days per week with moderate- or high-intensity loads for untrained individuals [1]. In the study by Watanabe [23], long-term intervention of ST-VLRE was performed with a training frequency of 2 days per week for untrained individuals. Despite the application of an extremely low-load, the low training frequency for ST-VLRE may be useful for enhancing exercise adherence because higher training frequencies may be considered a barrier for the participation and adherence to exercise [16]. Therefore, these findings suggest that ST-VLRE can be utilized as a universal resistance exercise protocol among various populations. However, the effect of ST-VLRE on cognitive function remains unknown.

We previously demonstrated that the level of increase in circulating lactate during acute aerobic exercise is associated with the degree of post-exercise IC improvements [25, 26], potentially caused by increasing cerebral lactate metabolism [25]. Watanabe et al. [23] reported that an increase in blood lactate induced by an acute bout of localized resistance exercise (i.e., knee extension exercise) was higher following ST-VLRE than normal VLRE. Based on the findings of our and other previous studies, we hypothesized that, despite the application of a very low-load (i.e., 30% 1-RM), ST-VLRE would effectively improve post-exercise IC, with greater improvements than that following VLRE. To test this hypothesis, we compared the effects of ST-VLRE and VLRE on post-exercise IC.

## 2 Methods

### 2.1 Subjects and ethics

Twenty healthy, young males (age: 21 ± 0 years, body height: 173.4 ± 1.2 cm, body weight: 67.4 ± 2.2 kg) participated in this study. Prior to this study, we calculated the required sample size utilizing an effect size (0.31), α-level of 0.05, and β-level of 0.2 (80% power), based on main outcome measure (i.e., the reverse-Stroop interference score) of our previous study [26]. The calculated necessary number of subjects was 14; therefore, the number of subjects recruited in this study was sufficient for ensuring statistical power and sensitivity. The subjects were recreationally active and participated in physical exercise (e.g., resistance exercise and/or aerobic exercise) for 2–4 h per week. They were free of any known neurological, cardiovascular, and pulmonary problems, as well as free from color-blindness and abnormal vision. All subjects were instructed to avoid strenuous physical activity in the 24 h prior to each experimental session. Each subject also abstained from food, caffeine, and alcohol for 12 h prior to each experiment, and was not taking any medications that may affect cognitive performance. All subjects provided written informed consent upon having the experimental procedures and potential risks described to them. The study was approved by the Ethics Committee of Ritsumeikan University and conducted according to the Declaration of Helsinki.

### 2.2 Experimental design

**Figure 1:**
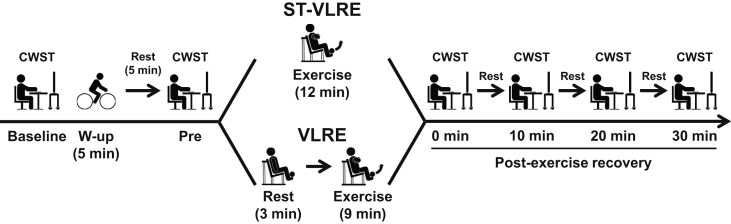
Experimental procedures of very low-intensity resistance exercise (VLRE) with slow movement and tonic force generation (ST; ST-VLRE) and VLRE sessions. Exercise load for both ST-VLRE and VLRE was set at 30% of one-repetition maximum. The bilateral knee extension exercise for both protocols was programmed for six sets with ten repetitions per set. The color-word Stroop task (CWST) was administered at baseline, before exercise (i.e., Pre), immediately after exercise, and every 10 min during the 30-min postexercise recovery period.

Experimental procedure of this study is presented in Figure 1. All subjects completed a familiarization visit where they practiced the three types of the color-word Stroop task (CWST), for each a minimum of 10 times until they achieved consistent scores, defined by an average reaction time over 5 practice trials of each CWST type for less than 500 ms, as described in our previous study [11]. During the familiarization visit, the subjects performed 1-RM measurement of bilateral knee extension.

The subjects were instructed to avoid strenuous physical activity within 24 h before experimental session. Each subject also abstained from food, caffeine and alcohol within 12 h before experiment session, and was not taking any medications that may affect cognitive function.

On the day of the experiment, the subjects practiced the three CWST types for each a minimum of five times before experimental session to minimize the learning effect. After the practice completed, the subjects rested for 5 min and then performed the baseline CWST.

Next, the subjects performed a warm-up exercise at 50 Watts for 5 min using a bicycle ergometer (Life Fitness; Schiller Park, IL, USA), similar to our previous studies [10, 11]. Heart rate (HR) and rating of perceived exertion (RPE) were recorded at the end during the warm-up exercise. After the warm-up exercise, the subjects rested for 5 min and then performed the pre-exercise CWST to confirm if the warm-up exercise affected IC and if IC before exercise session was reliable.

Thereafter, the subjects completed either ST-VLRE or VLRE. The CWST was performed again immediately after the completion of the exercise session and then repeated three times at 10-min intervals during the 30-min postexercise recovery period to evaluate the sustainable effects of post-exercise IC improvements [10, 25, 26, 27, 28, 29, 30, 31].

The cardiovascular and perceived exertion parameters and quadriceps femoris electromyographic (EMG) activity during exercise session were measured in every set to determine the levels of physiological and physiopsychological responses. Fingertip blood samples were collected immediately before all six CWSTs to measure blood glucose and lactate and to assess the effect of systemic metabolic conditions on IC. The felt arousal scale (FAS) and visual analogue scale (VAS) were measured immediately after all CWSTs to assess the effect of psychological conditions on IC.

### 2.3 Experimental protocols

All subjects completed both ST-VLRE and VLRE in a randomized and counterbalanced order. The ST-VLRE and VLRE were set at 30% 1-RM, based on a study by Watanabe et al. [23]. Both protocols were programmed with bilateral knee extension for six sets with ten repetitions per set using a leg extension machine (Life Fitness; Schiller Park, IL, USA). The ST-VLRE and VLRE were performed with slow (3-sec concentric, 3-sec eccentric, and 1-sec isometric actions with no rest between each repetition) and normal contractile speeds (1-sec concentric and 1-sec eccentric actions and 1-sec rests between each repetition), respectively, as in previous studies [20, 21, 22, 23, 24]. Rest intervals between sets for both protocols lasted 1 min. The two experimental sessions were performed at approximately the same time (±1 h) in the morning, as separated by 1 week.

### 2.4 1-RM

Subject's 1-RM was determined by the successful lift of the bilateral knee extension exercise on the familiarization visit day, as in our previous study [10, 11, 32]. The 1-RM value was used to determine the loads for the ST-VLRE and VLRE conditions. The 1-RM trial was designed using increments of 10 kg until 60–80% of the perceived maximum is achieved. Then, the load was gradually increased by 1–5 kg weights until lift fail, in which the subject was not able to maintain proper form or to completely lift the weight. The last acceptable lift with the highest possible load was defined as 1-RM. The mean 1-RM of bilateral knee extension in all subjects was 127 ± 6 kg. The mean load of 30% 1-RM for both ST-VLRE and VLRE were 38 ± 2 kg.

### 2.5 Cardiovascular parameters

HR was measured continuously via telemetry (RS400; Polar Electro Japan, Tokyo, Japan). Systolic blood pressure (SBP) and diastolic blood pressure (DBP) were measured using a mercury manometer (FC-110ST; Focal, Chiba, Japan). Mean arterial pressure (MAP) was calculated as [(SBP – DBP)/3 + DBP]. During exercise session, these cardiovascular parameters were collected immediately after each set, and the mean value of all six sets was calculated for analysis.

### 2.6 RPE

The Borg's RPE scale was measured to assess the perceived exertion expended during exercise, which ranges from 6 (no exertion) to 20 (maximal exertion) [33]. The Borg's category-ratio scale was also measured to assess the leg discomfort expended during exercise, which ranges from 0 (nothing at all) to 10 (very, very strong) [33]. During exercise session, RPE and leg discomfort were collected immediately after each set, and the mean value of all six sets was calculated for analysis.

### 2.7 Quadriceps femoris EMG

Prior to the application of electrodes, the subject's skin was shaved, abraded, and cleaned with alcohol to minimize skin impedance. EMG signals were recorded with surface electrodes from the quadriceps vastus lateralis, vastus medialis, and rectus femoris muscles. The EMG signals were amplified 1,000 times, band-pass filtered between 10 and 500 Hz, and sampled at 1,000 Hz (MQ-Air; Kissei Comtech, Nagano, Japan). The EMG activity of each quadriceps muscle during ST-VLRE and VLRE was quantified as the integration of the rectified EMG (iEMG) over 1 s. Thereafter, the iEMG were normalized to the highest iEMG (the average value over 1 s) that was obtained during the two trials of knee extension maximal voluntary contractions (MVC), which was measured after CWST was completed at the 30-min postexercise recovery period. Peak EMG activities of the three quadriceps femoris muscles were calculated every repetition per set and then averaged all ten repetitions for each set. The mean values of all six sets for each muscle were calculated for analysis.

### 2.8 Blood metabolites

Blood glucose and lactate levels were measured using a glucose (Medisafe FIT Blood Glucose Meter; Terumo, Tokyo, Japan) and lactate analyzer (Lactate Pro 2; Arkray, Kyoto, Japan), respectively.

### 2.9 CWST

The CWST was administered to determine IC [34]. The stimulus words were four color names (“RED”, “YELLOW”, “GREEN” and “BLUE”), and they were presented on a 98-inch display. The subjects were required to press the color-labeled key that corresponds to the text meaning (i.e., manual response). The three types of the CWST consisted of two color text tasks (i.e., congruent and incongruent tasks) and one control black text task (i.e., neutral task). The congruent task, which is a facilitate task, displayed the color names presented in the same-colored text. The neutral task, which is a control task, displayed the color names presented in black text. The incongruent task, which is an interference task, displayed the color names presented in a different-colored text. One trial of each type of the CWST consisted of 24 stimulus words. The three CWST types were repeated for each three trials. The intervals between each trial were set at 1 s. The reaction time and response accuracy for each trial were collected and the mean values of the three trials of each CWST type were calculated for analysis. The IC was assessed using the reverse-Stroop interference score, which is defined as the difference between reaction times of the neutral and incongruent tasks [10, 11, 25, 26, 27, 28, 29, 30, 31, 35]. The reverse-Stroop interference score is more appropriate for calculating the interference effect when measured by the manual response modality than the Stroop interference score, based on the findings from a previous study [36]. The reverse-Stroop interference score was calculated as [(reaction time of incongruent task – reaction time of neutral task)/reaction time of neutral task × 100] [36].

### 2.10 Psychological conditions

FAS was measured to assess the arousal during the CWST, which is a 6-point, single-item scale ranging from 1 (low-arousal) to 6 (high arousal) [37]. A high arousal represents “excitement” and a low arousal represents “relaxation”. VAS consisted of questions of three psychological types that assess mental fatigue, the ability to concentrate, and motivation for the CWST. Each VAS was labeled from 0 mm (i.e., not at all) to 100 mm (i.e., extremely). Subjects were verbally asked the simple question: “How did you feel about the three psychological variables during the CWST?” Then, the subjects drew lines consistent with their feelings for each psychological variable.

### 2.11 Statistical analysis

All data are expressed as the mean ± SEM, mean difference (MD), and 95% confidence interval (CI). Comparisons of HR and RPE at end-exercise during warm-up exercise in ST-VLRE and VLRE sessions were preformed using a paired Student's *t*-test. Similarly, mean values of cardiovascular (i.e., HR, SBP, DBP and MAP) and perceived exertion parameters (i.e., RPE and leg discomfort) and quadriceps EMG activities during the two exercise were compared using a paired Student's *t*-test. Changes in measured variables (i.e., blood metabolites, CWST-measured parameters, and physiological conditions) throughout two experimental sessions were analyzed using two-way (condition × time) repeated-measures analysis of variance. If the sphericity assumption was not met, Greenhouse-Geisser corrections were used. Specific differences were identified with a paired Student's *t*-test or Bonferroni *post-hoc* test. Statistical significance level was defined at *P* < 0.05. The Cohen's *d* effect size using the pooled SD was calculated to determine the magnitude of a difference in measured variables between conditions or among time points [38]. This effect size was interpreted as small (0.20–0.49), medium (0.50–0.79) and large (>0.80) [38]. Partial eta squared (*η*_p_^2^) values were determined as a measure of the effect size for main effects of condition and time or interaction effect. All statistical analyses were conducted using IBM SPSS software (Ver. 19.0, IBM Corp, NY, USA).

## 3 Results

### 3.1 Measured variables during warm-up and exercise sessions

The HR (89.9 ± 2.2 and 88.5 ± 2.1) and RPE (8.1 ± 0.4 and 8.1 ± 0.4) at the end-exercise during warm-up cycling did not differ significantly between ST-VLRE and VLRE sessions.

Mean values of cardiovascular and perceived exertion responses and quadriceps femoris EMG activities during ST-VLRE and VLRE were shown in Table 1. Mean HR during exercise was significantly higher for ST-VLRE than for VLRE (MD = 13.6, 95% CI: 7.8, 19.5, *P* < 0.001, *d* = 1.00). Although there was no significant difference for mean DBP during exercise between ST-VLRE and VLRE, mean SBP during exercise was significantly higher for ST-VLRE than for VLRE (MD = 7.3, 95% CI: 2.8, 11.8, *P* = 0.003, *d* = 0.60). Mean MAP showed a higher trend for ST-VLRE than for VLRE (MD = 2.9, 95% CI: 0.0, 5.8, *P* = 0.051, *d* = 0.40). Moreover, mean values of RPE and leg discomfort during exercise were significantly higher for ST-VLRE than for VLRE (MD = 3.4, 95% CI: 2.5, 4.3, *P* < 0.001, *d* = 1.81 for RPE; MD = 2.9, 95% CI: 2.4, 3.4, *P* < 0.001, *d* = 2.79 for leg discomfort). Furthermore, mean values of peak EMGs activities of all three quadriceps femoris muscles during exercise were significantly higher for ST-VLRE than for VLRE (MD = 11.1, 95% CI: 5.9, 16.4, *P* < 0.001, *d* = 0.79 for vastus lateralis; MD = 5.8, 95% CI: 0.6, 11.0, *P* = 0.032, *d* = 0.41 for vastus medialis; MD = 8.5, 95% CI: 2.3, 14.8, *P* = 0.010, *d* = 0.59 for rectus femoris).

**Table 1:** Mean values of cardiovascular and perceived exertion parameters and quadriceps femoris muscle electromyographic activities during very low-intensity resistance exercise (VLRE) with slow movement and tonic force generation (ST) and VLRE with normal speed (VLRE).

	ST-VLRE	VLRE
Heart rate, bpm	109.6 ± 3.8∗	96.0 ± 2.1
Systolic blood pressure, mmHg	132.1 ± 2.9∗	124.8 ± 2.6
Diastolic blood pressure, mmHg	64.3 ± 1.0	65.7 ± 1.3
Mean arterial pressure, mmHg	98.2 ± 1.6 ^*P*^^=0.051^	95.3 ± 1.7
Rating of perceived exertion	14.3 ± 0.4∗	10.9 ± 0.4
Leg discomfort	6.4 ± 0.3∗	3.4 ± 0.2
Peak electromyographic activity		
Vastus lateralis, % of MVC	66.6 ± 3.3∗	55.4 ± 3.0
Vastus medialis, % of MVC	55.8 ± 3.0∗	50.0 ± 3.4
Rectus femoris, % of MVC	45.2 ± 3.3∗	36.6 ± 3.2

Values are presented as Mean ± SEM. Mean values of measured variables were calculated as the average of all six sets. MVC: maximal voluntary contraction.

∗ Significant difference between ST-VLRE and VLRE.

### 3.2 Changes in blood metabolites throughout experimental session

**Figure 2:**
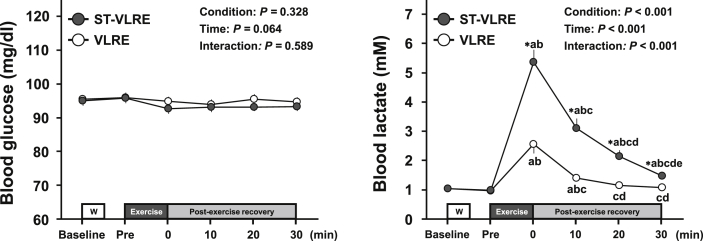
Changes in blood glucose and lactate levels throughout ST-VLRE and VLRE sessions. Values are presented as Mean ± SEM. ∗*P* < 0.001 vs. VLRE. ^a^*P* < 0.001 vs. Baseline; ^b^*P* < 0.05 vs. Pre; ^c^*P* < 0.05 vs. immediately after exercise (i.e., 0-min post-exercise recovery period); ^d^*P* < 0.05 vs. 10-min post-exercise recovery period; ^e^*P* < 0.05 vs. 20-min post-exercise recovery period.

Changes in blood metabolites throughout ST-VLRE and VLRE sessions are presented in Figure 2. Although blood glucose analysis revealed no significant main effect for condition and no significant interaction effect, a main effect for time showed a trend against significance (*F*_(5,95)_ = 2.17, *P* = 0.064, *η*_p_^2^ = 0.10). Blood lactate analysis revealed significant main effects for condition (*F*_(1,19)_ = 54.40, *P* < 0.001, *η*_p_^2^ = 0.74) and time (*F*_(1.19, 22.54)_ = 74.01, *P* < 0.001, *η*_p_^2^ = 0.80) and a significant interaction effect (*F*_(1.88, 35.79)_ = 50.27, *P* < 0.001, *η*_p_^2^ = 0.73). Blood lactate significantly increased immediately after both ST-VLRE and VLRE compared with that before each exercise (MD = 4.3, 95% CI: 2.9, 5.7, *P* < 0.001, *d* = 3.19 and MD = 4.4, 95% CI: 3.0, 5.8, *P* < 0.001, *d* = 3.24 vs. baseline and pre-exercise, respectively, for ST-VLRE; MD = 1.5, 95% CI: 0.6, 2.5, *P* = 0.001, *d* = 1.55 and MD = 1.6, 95% CI: 0.6, 2.6, *P* = 0.001, *d* = 1.61 vs. baseline and pre-exercise, respectively, for VLRE). Although the increased lactate following VLRE remained significant only until the 10-min post-exercise recovery period (MD = 0.4, 95% CI: 0.0, 0.7, *P* = 0.021, *d* = 0.95 and MD = 0.4, 95% CI: 0.1, 0.8, *P* = 0.011, *d* = 1.12 for baseline and pre-exercise, respectively, vs. 10-min post-exercise recovery period), the increased blood lactate following ST-VLRE remained significant throughout the 30-min post-exercise recovery period (MD = 0.4, 95% CI: 0.1, 0.7, *P* = 0.002, *d* = 1.29 and MD = 0.5, 95% CI: 0.2, 0.8, *P* < 0.001, *d* = 1.54 for baseline and pre-exercise, respectively, vs. 30-min post-exercise recovery period). Blood lactate levels throughout the 30-min post-exercise recovery period were significantly higher for ST-VLRE than for VLRE (MD = 0.4, 95% CI: 0.2, 0.6, *P* < 0.001, *d* = 1.25 for 30-min post-exercise recovery period).

### 3.3 Changes in the CWST-measured IC throughout experimental session

Changes in reaction times and response accuracies on three types of the CWST throughout ST-VLRE and VLRE sessions are summarized in Table 2. Analyses of reaction times for congruent and neutral tasks revealed significant main effects for time (*F*_(5,95)_ = 4.80, *P* = 0.001, *η*_*p*_^*2*^ = 0.20 for congruent task; *F*_(2.09, 39.72)_ = 4.86, *P* = 0.012, *η*_p_^2^ = 0.20 for neutral task). Congruent reaction time immediately after ST-LRE was significantly shorter than that of 30 min after this exercise (MD = -534.8, 95% CI: -1047.0, -22.6, *P* = 0.036, *d* = 0.45). Neutral reaction time 10 min after ST-VLRE was significantly shorter than that before exercise (MD = -319.2, 95% CI: -613.9, -24.5, *P* = 0.027, *d* = 0.22 vs. pre-exercise). By contrast, reaction times of congruent and neutral tasks did not differ significantly among all time points throughout VLRE sessions. Incongruent task analysis revealed a significant main effect for time (*F*_(2.70, 51.38)_ = 21.38, *P* < 0.001, *η*_p_^2^ = 0.53) and a significant interaction effect (*F*_(5,95)_ = 3.44 *P* = 0.007, *η*_p_^2^ = 0.15). Incongruent reaction time significantly shortened immediately after both ST-VLRE and VLRE compared with that before each exercise (MD = -683.20, 95% CI: -1067.3, -299.1, *P* < 0.001, *d* = 0.41 and MD = -649.6, 95% CI: -1075.3, -223.9, *P* = 0.001, *d* = 0.41 vs. baseline and pre-exercise, respectively, for ST-VLRE; MD = -481.2, 95% CI: -803.3, -159.0, *P* = 0.001, *d* = 0.29 vs. pre-exercise for VLRE). The shortened incongruent reaction time following both protocols remained significant until the 20-min post-exercise recovery period (MD = -767.9, 95% CI: -1199.8, -335.9, *P* < 0.001, *d* = 0.51 and MD = -734.3, 95% CI: -1091.7, -376.9, *P* < 0.001, *d* = 0.51 for baseline and pre-exercise, respectively, vs. 20-min post-exercise recovery period for ST-VLRE; MD = -264.9, 95% CI: -526.7, -3.0, *P* = 0.046, *d* = 0.16 for pre-exercise vs. 20-min post-exercise recovery period for VLRE). The incongruent reaction times at 10 and 20 min after exercise were significantly shorter for ST-VLRE than for VLRE (MD = -400.8, 95% CI: -771.1, -30.6, *P* = 0.035, *d* = 0.26 for 10-min post-exercise recovery period; MD = -514.6, 95%CI: -896.1, -133.1, *P* = 0.011, *d* = 0.35 for 20-min post-exercise recovery period). Analyses of response accuracies for all three CWST tasks revealed no significant main effects for time and condition and no significant interaction effects.

**Table 2:** Changes in reaction times and response accuracies of the three color-word Stroop tasks throughout ST-VLRE and VLRE sessions.

	Time points						*P* values		
	Baseline	Pre-EX	Post-EX 0	Post-EX 10	Post-EX 20	Post-EX 30	Condition	Time	Interaction
**Reaction time (msec)**									
*Congruent task*									
ST-VLRE	8618 ± 304	8575 ± 282	8171 ± 250	8279 ± 267	8483 ± 272	8706 ± 281^c^	0.081	0.001	0.247
VLRE	8575 ± 353	8698 ± 288	8529 ± 321	8505 ± 338	8838 ± 324	8936 ± 285			
*Neutral task*									
ST-VLRE	9193 ± 350	9173 ± 332	8894 ± 317	8854 ± 303^b^	8949 ± 268	9089 ± 295	0.430	0.012	0.919
VLRE	9205 ± 397	9199 ± 337	9011 ± 321	8974 ± 340	9068 ± 341	9223 ± 299			
*Incongruent task*									
ST-VLRE	10146 ± 380	10112 ± 353	9462 ± 359 ^ab^	9332 ± 306 ∗^ab^	9378 ± 282 ∗^ab^	9956 ± 332 ^cde^	0.130	<0.001	0.007
VLRE	10157 ± 433	10157 ± 385	9676 ± 355^b^	9732 ± 369^b^	9892 ± 377^b^	10152 ± 336^c^			
**Response accuracy (%)**									
*Congruent task*									
ST-VLRE	96 ± 1	97 ± 1	97 ± 1	96 ± 1	95 ± 1	95 ± 1	0.471	0.368	0.461
VLRE	96 ± 1	95 ± 1	96 ± 1	96 ± 1	96 ± 1	95 ± 1			
*Neutral task*									
ST-VLRE	96 ± 1	96 ± 1	96 ± 1	96 ± 1	95 ± 1	95 ± 1	0.211	0.674	0.317
VLRE	96 ± 1	96 ± 1	96 ± 1	96 ± 1	95 ± 1	96 ± 1			
*Incongruent task*									
ST-VLRE	95 ± 1	95 ± 1	96 ± 1	95 ± 1	96 ± 1	96 ± 1	0.241	0.875	0.883
VLRE	95 ± 1	95 ± 1	95 ± 1	95 ± 1	95 ± 1	95 ± 1			

Values are presented as Mean ± SEM. Pre-EX; before exercise, Post-EX 0; immediately after exercise, Post-EX 10; 10-min at post-exercise recovery period, Post-EX 20; 20-min at post-exercise recovery period, Post-EX 30; 30-min at post-exercise recovery period.

**Figure 3:**
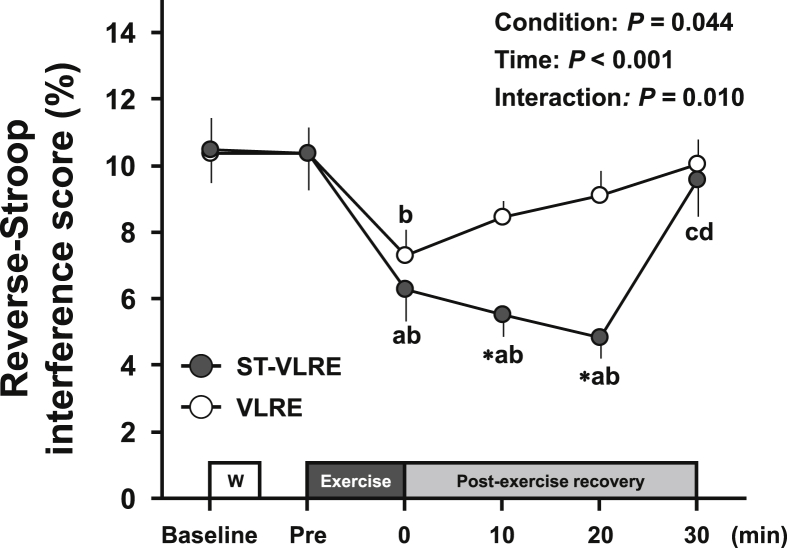
Changes in the reverse-Stroop interference scores throughout ST-VLRE and VLRE sessions. Values are presented as Mean ± SEM. ∗*P* < 0.001 vs. VLRE; ^a^*P* < 0.001 vs. Baseline; ^b^*P* < 0.05 vs. Pre; ^c^*P* < 0.05 vs. 0-min post-exercise recovery period; ^d^*P* < 0.05 vs. 10-min post-exercise recovery period.

Changes in the reverse-Stroop interference score throughout ST-VLRE and VLRE sessions are presented in Figure 3. The reverse-Stroop interference score analysis revealed a significant main effects for condition (*F*_(1,19)_ = 4.67, *P* = 0.044, *η*_p_^2^ = 0.20) and time (*F*_(5,95)_ = 11.89, *P* < 0.001, *η*_p_^2^ = 0.39) and a significant interaction effect (*F*_(5,95)_ = 3.21, *P* = 0.010, *η*_p_^2^ = 0.15). The reverse-Stroop interference score significantly decreased immediately after both ST-VLRE and VLRE compared with that before each exercise (MD = -4.2, 95% CI: -8.0, -0.5, *P* = 0.019, *d* = 1.01 and MD = -4.1, 95% CI: -8.2, 0.0, *P* = 0.050, *d* = 1.07 vs. baseline and pre-exercise, respectively, for ST-VLRE; MD = -3.1, 95% CI: -5.9, -0.2, *P* = 0.030, *d* = 0.73 vs. pre-exercise for VLRE). The decreased reverse-Stroop interference score remained significant until 20 min after ST-VLRE, but not after VLRE (MD = -5.7, 95% CI: -8.6, -2.7, *d* = 1.59 *P* < 0.001 and MD = -5.5, 95% CI: -8.0, -3.1, *P* < 0.001, *d* = 1.76 for baseline and pre-exercise, respectively, vs. 20-min post-exercise recovery period).

### 3.4 Changes in psychological conditions for the CWST throughout experimental session

Changes in psychological conditions for the CWST throughout ST-VLRE and VLRE sessions are shown in Table 3. Arousal analysis revealed a significant main effect for time (*F*_(2.57, 48.90)_ = 19.23, *P* < 0.001, *η*_p_^2^ = 0.50) and a significant interaction effect (*F*_(3.16, 60.12)_ = 7.20, *P* < 0.001, *η*_p_^2^ = 0.28). Arousal significantly increased immediately after both ST-VLRE and VLRE compared with that before each exercise (MD = 1.5, 95% CI: 0.7, 2.3, *P* < 0.001, *d* = 2.10 and MD = 1.5, 95% CI: 0.8, 2.2, *P* < 0.001, *d* = 2.00 vs. baseline and pre-exercise, respectively, for ST-VLRE; MD = 0.8, 95% CI: 0.2, 1.4, *P* = 0.006, *d* = 0.98 and MD = 0.6, 95% CI: 0.0, 1.1, *P* = 0.030, *d* = 0.72 vs. baseline and pre-exercise, respectively, for VLRE). The increased arousal remained significant until 10 min after ST-VLRE (MD = 0.8, 95% CI: 0.2, 1.3, *P* = 0.006, *d* = 1.23 and MD = 0.8, 95% CI: 0.3, 1.2, *P* = 0.001, *d* = 1.15 vs. baseline and pre-exercise, respectively), but not after VLRE. Arousal immediately after exercise was significantly higher for ST-VLRE than for VLRE (MD = 0.7, 95% CI: 0.3, 1.0, *P* = 0.002; *d* = 0.77). Mental fatigue analysis revealed a significant effect for time (*F*_(3.46, 65.67)_ = 26.43, *P* < 0.001, *η*_p_^2^ = 0.58) and a significant interaction effect (*F*_(3.28, 62.26)_ = 4.45, *P* = 0.005, *η*_p_^2^ = 0.19). Mental fatigue significantly increased immediately after both ST-VLRE and VLRE compared with that before each exercise (MD = 39.9, 95% CI: 19.4, 60.3, *P* < 0.001, *d* = 1.93 and MD = 32.6, 95%CI: 14.6, 50.5, *P* < 0.001, *d* = 1.41 vs. baseline and pre-exercise, respectively, for ST-VLRE; MD = 18.0, 95%CI: 5.5, 30.4, *P* = 0.002, *d* = 0.89 vs. baseline for VLRE). The increased mental fatigue remained significant throughout the 30-min post-exercise recovery period for ST-VLRE (MD = 21.8, 95%CI: 6.2, 37.3, *P* = 0.002, *d* = 1.07 and MD = 14.5, 95%CI: 3.0, 25.9, *P* = 0.007, *d* = 0.63 for baseline and pre-exercise, respectively vs. 30-min post-exercise recovery period). By contrast, mental fatigue levels only 10 and 30 min after VLRE were significantly higher than that before this exercise (MD = 15.0, 95% CI: 0.6, 29.4, *P* = 0.037, *d* = 0.70 vs. baseline for 10-min post-exercise recovery period; MD = 17.3, 95% CI: 2.9, 31.7, *P* = 0.010, *d* = 0.77 vs. baseline for 30-min post-exercise recovery period). Mental fatigue immediately after exercise was significantly higher for ST-VLRE than for VLRE (MD = 22.4, 95% CI: 9.1, 35.6, *P* = 0.002, *d* = 0.96). Although motivation analysis revealed no significant main effects for condition and no significant interaction effect, a trend against significance was observed for a main time effect (*F*_(2.01, 38.24)_ = 2.80, *P* = 0.073, *η*_p_^2^ = 0.13). Concentration analyses revealed no significant main effects for condition and time and no significant interaction effect.

**Table 3:** Psychological conditions for the color-word Stroop task throughout ST-VLRE and VLRE sessions.

	Time points						*P* values		
	Baseline	Pre-EX	Post-EX 0	Post-EX 10	Post-EX 20	Post-EX 30	Condition	Time	Interaction
Arousal									
ST-VLRE	2.8 ± 0.1	2.8 ± 0.1	4.3 ± 0.2 ∗^ab^	3.6 ± 0.2 ^abc^	3.2 ± 0.2 ^cd^	3.0 ± 0.1 ^cd^	0.491	<0.001	<0.001
VLRE	2.9 ± 0.2	3.1 ± 0.2	3.7 ± 0.2 ^ab^	3.2 ± 0.2	3.2 ± 0.2	3.2 ± 0.2			
Mental fatigue (mm)									
ST-VLRE	21.8 ± 3.9	29.1 ± 5.1	61.6 ± 5.2 ∗^ab^	42.8 ± 4.1 ^ac^	45.2 ± 4.7 ^abc^	43.5 ± 5.1 ^ab^	0.088	<0.001	0.005
VLRE	21.3 ± 3.6	29.3 ± 5.6	39.3 ± 5.2^a^	36.3 ± 5.7^a^	33.7 ± 5.6	38.6 ± 6.1^a^			
Concentration (mm)									
ST-VLRE	58.7 ± 4.5	55.5 ± 3.8	68.1 ± 4.6	66.7 ± 4.5	59.1 ± 4.3	54.0 ± 4.2	0.104	0.093	0.200
VLRE	65.6 ± 3.4	64.8 ± 3.1	67.4 ± 3.5	63.0 ± 4.7	61.0 ± 3.9	60.3 ± 4.8			
Motivation (mm)									
ST-VLRE	70.4 ± 3.8	67.8 ± 3.2	71.3 ± 3.9	69.2 ± 4.3	63.3 ± 4.2	65.5 ± 4.3	0.669	0.073	0.810
VLRE	70.4 ± 3.6	70.2 ± 3.7	72.9 ± 3.5	68.2 ± 4.8	65.5 ± 5.0	65.0 ± 5.3			

Values are presented as Mean ± SEM. ∗*P* < 0.05 vs. VLRE; ^a^*P* < 0.05 vs. Baseline; ^b^*P* < 0.05 vs. Pre-EX; ^c^*P* < 0.05 vs. Post-EX 0 min; and ^d^*P* < 0.05 vs. Post-EX 10 min.

## 4 Discussion

The primary finding of this study was that, although IC (i.e., reverse-Stroop interference score) significantly improved immediately after both ST-VLRE and VLRE compared with that before each exercise, the improvement remained significant until 20 min after ST-VLRE; however, not after VLRE. Furthermore, the degree of IC improvements throughout the 30-min post-exercise period was significantly greater for ST-VLRE than for VLRE, as assessed based on a significant interaction effect. These findings suggest that ST-VLRE may be a more effective protocol for improving IC than VLRE.

In this study, we found significant improvement (i.e., immediate effect) in immediate post-exercise IC for both ST-VLRE and VLRE. However, the significant IC improvement was sustained for 20 min after ST-VLRE, whereas it was not observed after VLRE. Our previous studies suggested that, in addition to the immediate effect, the sustainable effect (i.e., duration of significant improvements in post-exercise IC) may be an important factor for understanding the potential impact on post-exercise IC improvements among various aerobic exercise protocols [10, 25, 26, 27, 28, 29, 30, 31]. Several previous studies examined the sustainable effect of some resistance exercise protocols on post-exercise IC improvements [7, 9, 10]. Of these previous studies, in the same population (i.e., healthy young males) as the present study, we recently determined that significant improvement in post-exercise IC was sustained until 20 min after both high-volume LRE (i.e., consisted of 35% 1-RM, 20 repetitions/set, and 6 sets) and volume-matched HRE (i.e., consisted of 70% 1-RM, 10 repetitions/set, and 6 sets), with large effect size (all *d*s = 0.80 to 1.05 vs. baseline and pre-exercise for both protocols) [10], which was similar to that after ST-VLRE (all *d*s = 1.01 to 1.76 vs. baseline and pre-exercise). Therefore, despite lesser exercise volume and intensity, ST-VLRE can obtain a degree of post-exercise IC improvement similar to the high-volume or high-intensity protocol of resistance exercise.

In comparison with aerobic exercise, the sustainable effect of ST-VLRE may be similar to that of moderate-intensity exercise (i.e., 60% of peak oxygen consumption) lasting 40 min, which sustained significant improvement in post-exercise IC, with large effect size (*d* = 1.59 vs. pre-exercise), until 20 min after this exercise in healthy young males [30]. The ST-VLRE employed in the present study was completed in 12 min, suggesting that it could be programmed in a more time-efficient manner. Exercise duration is an important factor in regulating exercise adherence [16], with a shorter duration is possibly useful for improving the adherence. Therefore, ST-VLRE may hold underlying potential for enhancing adherence to resistance exercise in various populations.

The results of this study showed that an increase in blood lactate induced by exercise was greater after ST-VLRE than after VLRE. This finding corroborates the result of a study by Watanabe et al. [23]. The circulating lactate is utilized as an important energy substrate instead of glucose during exercise in the human brain [39]. Our previous studies demonstrated that level of increase in blood lactate during aerobic exercise is associated with the degree of post-exercise IC improvements [25, 26], potentially caused by increasing cerebral lactate metabolism [25]. The findings of our studies may contribute to interpreting the present finding by showing that the degree of post-exercise IC improvements was greater for ST-VLRE than for VLRE.

This study determined greater EMG activities of the quadriceps femoris muscles during ST-VLRE than those during VLRE. Watanabe et al. [23] reported that the EMG activity of the vastus lateralis was greater at the last set than at the first set during ST-VLRE, whereas no such difference was observed during VLRE. The increased muscle activity during resistance exercise may be associated with cerebral neural activation [40, 41]. The neural activation in the brain, particularly in the dorsolateral prefrontal cortex, is considered to be likely the most prominent determinant of cognitive performances, including IC, because it is increased during the CWST [42, 43, 44, 45, 46]. Previous studies reported a positive relationship between higher dorsolateral prefrontal cortex activity and better CWST-measured IC [43, 45]. Furthermore, previous studies demonstrated that an increase in the dorsolateral prefrontal cortex activity following exercise is related to an improved CWST-measured post-exercise IC [42, 44, 46]. Therefore, the greater neural activation in the exercising muscles induced by ST-VLRE, compared to VLRE, may help our understanding of the difference in the degree of post-exercise IC improvements, potentially via cerebral neural activation, between the two protocols.

Despite the positive effects on cognitive function and cerebral neural activation, the higher EMG activity of the exercising muscles appears to be a risk factor for injuries of the musculoskeletal system, especially of the skeletal muscle, among some populations [47]. Nevertheless, the EMG activities of the quadriceps femoris muscles during ST-VLRE (i.e., 67 % of MVC for vastus lateralis, 56 % of MVC for vastus medialis, 45 % of MVC for rectus femoris) were lower than those during both high-volume LRE and HRE (i.e., 81 and 101 % of MVC, respectively, for vastus lateralis; 79 and 98 % of MVC, respectively, for lateralis medialis; 65 and 103 % of MVC, respectively, for rectus femoris) obtained in our previous study [10]. In addition to potential benefit to prevent the skeletal muscle-related injury, the application of a very low-load involved in performing resistance exercise may lower the risk of injuries of the joint and connective tissues [47, 48], because of lower mechanical stresses to these tissues than that of the application of high-loads [13]. In particular, this is useful in reducing pain and damage to these tissues induced by resistance exercise in some individuals, including patients with degenerative joint diseases (e.g., osteoarthritis) [14]. Therefore, ST-VLRE may be an effective protocol for improving IC, with a lower risk of injuries of the musculoskeletal system, in various populations.

In this study, arousal immediately after exercise was greater for ST-VLRE than for VLRE. Byun et al. [42] reported that an increase in arousal induced by exercise is related to post-exercise IC improvement and cerebral neural activation. These findings of the present and previous studies may contribute to our understanding of the results of post-exercise IC improvements obtained in the present study.

This study determined greater cardiovascular responses (i.e., increased HR and blood pressure variables) during ST-VLRE than those during VLRE. Of these parameters, mean SBP immediately after each set of exercise session, as measured by the means of standard telemetry, was significantly greater for ST-VLRE than for VLRE. A trend against such significance was observed for mean MAP. Tanimoto et al. [21] reported a greater decrease in oxygenation level of the vastus lateralis during knee extension resistance exercise for LRE (i.e., 50% 1-RM) with ST than that for LRE and HRE (i.e., 80% 1-RM) with normal speeds. Muscle hypoxia (i.e., decreased oxygenation level of the exercising muscle) during exercise contributes to blood pressure elevation via the chemoreflex [49, 50]. Thus, greater elevations in SBP and MAP during ST-VLRE than those during VLRE observed in the present study may be attributed to accelerated hypoxia of the exercising muscles (i.e., the knee extensor muscles). However, Watanabe et al. [23] reported that SBP during knee extension resistance exercise, as measured by the means of arterial tonometry, did not differ significantly between ST-VLRE and VLRE. This discrepancy between the findings of the present and previous studies may be due to the measurement method and timing. Although this discrepancy is controversial, our findings suggest that ST-VLRE may be a higher risk factor for cardiovascular events than VLRE among some populations. Nevertheless, the present study determined that mean DBP immediately after each set of exercise session did not differ significantly between ST-VLRE and VLRE. Moreover, mean MAP immediately after each set during ST-VLRE (i.e., 98 mmHg) was lower than the MAP after some aerobic exercise protocols, such as moderate-intensity continuous exercise protocols (e.g., 106–110 mmHg as 10-40-min durations [28, 30, 31]), obtained in our previous studies [25, 26, 27, 28, 29, 30, 31]. Furthermore, blood pressure responses during ST-VLRE may be lower than those during normal HRE obtained in our previous studies [10, 21]. Therefore, the blood pressure response during ST-VLRE cannot be considered excessive.

Long-term intervention using HRE is known to deteriorate vascular function [51], which may be, at least partially, associated with excessive elevations in blood pressure induced by acute exercise bouts throughout this intervention period [52, 53]. By contrast, Okamoto et al. [54] reported that long-term intervention using LRE, comprising 40% 1-RM with ST, improved vascular function. This may be attributed to an increase in nitric oxide production, potentially through repeated cycles of hypoxia (i.e., decreased oxygenation level during exercise) and hyperoxia (increased oxygenation level after exercise) exposed to the exercising muscles during acute exercise bouts of LRE with ST [21, 22, 24]. In summary, ST-LRE may be an effective protocol for improving IC, with some potential benefits on cardiovascular responses.

In this study, mean values of perceived exertion parameters (i.e., RPE and leg discomfort) during exercise were greater for ST-VLRE than for VLRE. The increase in perceived exertion response during exercise is considered a barrier to participation and adherence to exercise [16]. Nevertheless, RPE during ST-VLRE (i.e., approximately 14) was lower than that during both high-volume LRE and HRE (i.e., approximately 15 for both protocols). Therefore, ST-LRE may have potential benefits regarding resistance exercise-induced perceptual responses.

Another finding of this study was that IC significantly improved immediately after normal VLRE compared to that before this exercise, with median or large effect size (*d* = 0.83 and 0.73 vs. baseline and pre-exercise, respectively). Our previous study determined that LRE using 40% 1-RM improved IC immediately after exercise in healthy young males, with median effect size (*d* = 0.75) [11]. Hence, despite a lower-load, the immediate effect on post-exercise IC for VLRE may be greater than or similar to that of the LRE protocol. Although the number of repetitions per set and number of sets were same between the two protocols, the length of the rest interval between sets was shorter during VLRE than LRE (i.e., 1 min vs. 3 min, respectively). The inter-set rest interval length is known to be an important variable for creating an effective resistance exercise program [17]. Previous studies determined that protocols with short inter-set rest interval, such as with VLRE, may be more useful for enhancing muscle size and strength adaptations induced by long-term resistance exercise than protocols with long inter-set rest interval [55, 56]. This positive effect may be due to increased levels of by-products, such as lactate, during an acute bout of this type of protocol [56, 57]. Therefore, because of the close relationship between lactate metabolism and cognitive function during acute exercise [25, 26], VLRE with short inter-set rest intervals may be an option for creation of effective resistance exercise protocols that improve cognitive function, including IC.

A major limitation of this study that although this study recruited healthy young males, effective resistance exercise programs to improve cognitive function are generally more important for older individuals and patients with chronic diseases than for healthy young individuals. Previous studies determined that long-term interventions of resistance exercise protocols, including VLRE, with ST increased muscle size and strength effectively in older individuals and patients with chronic diseases [20, 23, 24]. To further popularize ST-VLRE in the clinical setting, further studies are needed to determine the effects of ST-VLRE on post-exercise IC improvements in these populations.

## 5 Conclusion

This study determined that post-exercise IC improvements were greater for ST-VLRE than for VLRE. These present findings suggest that, despite the application of a very low-load, ST-VLRE can effectively improve post-exercise IC. Therefore, ST-VLRE may be an effective resistance exercise protocol for improving cognitive function, including IC, in various populations.

## Declarations

### Author contribution statement

Kento Dora and Tadashi Suga: Conceived and designed the experiments; Performed the experiments; Analyzed and interpreted the data; Wrote the paper.

Keigo Tomoo, Takeshi Sugimoto and Ernest Mok: Performed the experiments; Analyzed and interpreted the data.

Hayato Tsukamoto, Shingo Takada, Takeshi Hashimoto and Tadao Isaka: Analyzed and interpreted the data.

### Funding statement

This work was supported the by Center of Innovation Program from 10.13039/501100002241Japan Science and Technology Agency (#JPMJCE1306 to Tadashi Suga and Tadao Isaka,; #JPMJCE1301 to Shingo Takada).

### Data availability statement

Data will be made available on request.

### Declaration of interests statement

The authors declare no conflict of interest.

### Additional information

No additional information is available for this paper.
